# Are you dense? The implications and imaging of the dense breast

**DOI:** 10.4102/sajr.v22i2.1356

**Published:** 2018-08-23

**Authors:** Jacqueline S. Smilg

**Affiliations:** 1 Evolutionary Studies Institute, University of the Witwatersrand, South Africa; 2 Department of Radiation Sciences, University of the Witwatersrand, South Africa; 3 Department of Diagnostic Radiology, Charlotte Maxeke Johannesburg Academic Hospital, Johannesburg, South Africa

## Abstract

Mammography relies on a visual interpretation of imaging results that is often confounded by dense breast tissue. Dense tissue affects the ability and accuracy with which the radiologist is able to detect cancer. Dense tissue may mask the presence of a breast cancer, and breast density is well recognised as an independent risk factor for the development of breast cancer. In the dense breast, detected cancers tend to be larger, more often lymph node positive and of a higher stage than those diagnosed in fatty tissue. The incidence of tumour multifocality and multicentricity is higher, decreasing the chances for breast conserving treatment. The literature convincingly supports the use of supplemental imaging modalities in women who present with increased breast density. There are clear advantages and disadvantages to each set of diagnostic imaging tests. However, there is no simple, cost-effective solution for women with dense breasts to obtain a definitive detection status through imaging. Suggestions are put forward as to what supplemental imaging choices should be included for the imaging of the dense breast with reference to the current South African setting. Use of supplemental screening modalities should be tailored to individual risk assessment. In a resource-constrained environment, international recommendations may need to be adjusted.

## Introduction and review

Breast cancer is the most common female cancer in South Africa and is a leading cause of death amongst South African women.^1^ The increasing incidence of breast cancer is a major health concern with 19.4 million women aged 15 years and older at risk of contracting the disease.^1^ Breast cancer is the most common form of cancer to affect women in South Africa, and in 2013, it was responsible for 20.8% of female cancers and more than 10% of the entire cancer burden.^1^

Breast cancer screening aims to detect the disease early and thereby reduce mortality from breast cancer.^2^ Estimating an individual woman’s absolute risk for breast cancer is essential when decisions are being made about screening and preventive recommendations.^3^

The risk factors that are identified to play a role in predicting an individual’s potential breast cancer risk include current age, age at menarche, age at first live birth, number of previous breast biopsies and first-degree relatives with breast cancer.^4^ Several studies have assessed the contribution of adding a measure of mammographic density to breast cancer risk prediction models.^3,5^ Mammographic density is one of the strongest risk factors for breast cancer, with a high population attributable risk.^6^

The density of breast tissue is that portion of the breast that is composed of glandular and connective tissue. A dense breast is one in which there is more tissue than fat and this type of tissue is more common in younger women. About 40% of women over the age of 40 years have dense breasts.^7^ The breasts tend to become more replaced by fat as the glands involute after menopause. Dense breast tissue reduces the effectiveness of mammography and increases the risk for developing breast cancer.

**FIGURE 1:**
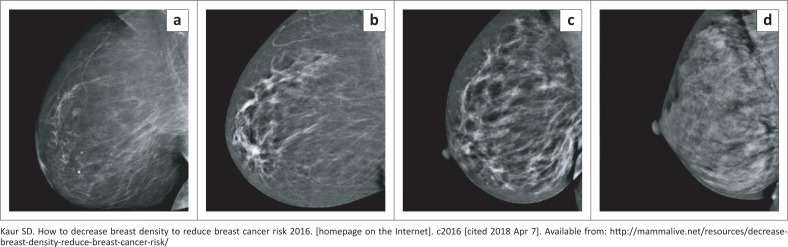
Mammographic demonstration of Breast Imaging Reporting and Data System (BI-RADS) breast density categories: (a) < 25% density – fatty breast tissue; (b) < 50% density – scattered density; (c) > 50% density – heterogeneously dense; and (d) > 75% density – extremely dense.

The Breast Imaging Reporting and Data System (BI-RADS) breast density categories are used in mammographic reports to indicate the degree of mammographic breast density (Figure 1)^8^:

- The breasts are almost entirely fatty.
- There are scattered areas of fibroglandular density.
- The breasts are heterogeneously dense.
- The breasts are extremely dense.

The last two categories are considered ‘dense’. When considering women aged in their early 40s, about 13% have extremely dense breasts and 44% have heterogeneously dense breasts. By the early 70s of age, 2% have extremely dense breasts and 24% have heterogeneously dense breasts.^7^ The fifth edition of BI-RADS, when compared to the fourth edition, places more emphasis on the masking effects of breast density. This edition specifies that when there are regions of sufficient density to obscure small masses, the mammogram should be categorised as heterogeneously dense rather than with scattered fibroglandular tissue, even if the overall volume of density would not typically place that study in the heterogeneous category.^9^

Because of inherent inter- and intra-reader variability of BI-RADS density classification, computer-based methods have been developed to improve consistency. Several automated density programmes have demonstrated high reproducibility^10^ and correlation with volumetric density as measured by magnetic resonance imaging (MRI).^11^

Many studies have concluded that there is at least a moderate association of mammographic breast density and the risk of breast cancer.^12^ Extremely dense tissue poses a four to six times increased likelihood of developing breast cancer when compared to the risk with fatty involuted tissue and twice the likelihood when compared to scattered fibroglandular density type.^12^

Women with dense breast tissue on mammogram are at increased risk for interval cancer (cancer that presents because of symptoms during the time between regular screening) because of the mammographic challenges and limitations of cancer detection for women with dense tissue. Boyd et al.^13^ reported that women with extremely dense tissue were 17 times more likely to have an interval cancer than women with fatty involuted tissue. Interval cancers represented 15.7% of cancers in extremely dense breasts compared with 4.5% of cancers in fatty tissue.^14^ It has been reported that cancers detected in dense tissue are larger, more likely to be lymph node positive and of a higher stage than in women without dense tissue, more often multifocal or multicentric, and mastectomy is more often performed.^15,16,17^ Dense breast tissue increases the risk of breast cancer and impairs detection of non-calcified cancers on mammography, which can result in a more advanced stage at diagnosis.

Digital mammography improves cancer detection in dense tissue compared with film-screen mammography^18^; however, supplemental screening in addition to mammography may be indicated for women with dense tissue allowing for earlier detection of cancers in the dense breast.

Supplemental screening can include the following:

- **Digital breast tomosynthesis (DBT):** Many studies have shown that there is an improvement in invasive cancer detection with DBT, but fewer studies have addressed its performance in differing density categories. Ciatto et al.^19^ showed an incremental cancer detection rate because of DBT of 2.8 per 1000 mammograms with fatty or scattered fibroglandular tissue and 2.5 per 1000 in dense tissue. Digital breast tomosynthesis improves cancer detection compared to standard digital mammography in women with heterogeneously dense breasts but may be less effective in women with extremely dense breasts. A single centre study found that DBT reduced the interval cancer rate across all densities but most studies lack sufficient follow-up to substantiate this.^20^
- **Ultrasound:** Ultrasound improves detection of early stage invasive breast cancer and is the most frequently used supplemental screening modality. Data from many studies have revealed an increase in the rate of cancer detection each year when supplemental ultrasonography was utilised.^21,22,23,24,25,26^ The adjunct screening with tomosynthesis or ultrasound in mammography-negative dense breasts (ASTOUND) trial is the first published prospective trial directly comparing sonar and 3D mammography after negative 2D mammography in dense tissue.^24^ Hesitation still exists in implementing routine, supplemental ultrasonography screening despite the data from the aforementioned studies. Using handheld 2D ultrasonography to detect small masses is labour intensive. Operator variability, shortages of trained personnel and reductions in radiologist efficiency for image acquisition all contribute to the widespread discouragement for whole-breast surveys.^26^ In order to combat some of these challenges, 3D automated whole-breast ultrasonography has been introduced as an alternative modality. Multiple studies substantiate that supplemental breast ultrasonography – whether 2D handheld or 3D automated whole-breast ultrasonography – can improve rates of cancer detection.^21,22,23,24,25,26^ Ultrasonography is in common use for diagnostic breast imaging, but its role in screening remains unclear. Studies utilising screening ultrasonography demonstrate its capability for detecting invasive malignancies in dense breasts at small sizes and localised stages that could potentiate an increase in breast cancer survival rate; however, more studies are needed to determine the impact on mortality.^26^ The best indications for screening ultrasonography in dense breasts may be for women with intermediate risk or in those women at high risk but with a contraindication to MRI.^22^
- **MRI:** The evidence supporting MRI screening of the breast continues to evolve. A multicentre trial by Sardanelli et al.^27^ determined MRI to be more sensitive (91%) than clinical breast examination (18%), mammography (50%), ultrasonography (52%) or mammography plus ultrasonography (63%). In addition, 31% of cancers were detected by MRI alone. Many other studies have shown similar results.^22,26^ MRI is recommended for supplemental screening in women at high risk of breast cancer regardless of breast density, but cost and availability limit its use for general screening. Although cost, patient tolerance and accessibility are major detriments to using breast MRI to screen women with the sole indication of dense breast tissue, some investigators are developing abbreviated examinations that show promising results.^28^
- **Contrast enhanced digital mammography (CEDM):** This technology is being explored for screening. Based on diagnostic work in women with known cancer,^29,30^ sensitivity is likely comparable to MRI and specificity may be higher. In the diagnostic setting, CEDM has been demonstrated to be superior to standard mammography in women with dense breasts.^31,32^
- **Molecular breast imaging (MBI):** Studies using ^99m^Tc-sestamibi have been performed for supplemental screening of women with dense breasts.^33^ Studies are typically time consuming and the typical dose of about 740 MBq (20 mCi) has been considered excessive for use as a screening test.^34^ The radiation exposure to the whole body, and not just the breast, is five times that of digital mammography, and twice that of combination digital mammography and DBT. These facts, taken in conjunction with the knowledge that there is no data on interval cancer rates, make it unlikely that MBI will be implemented in common practice.^35^

## Conclusion

The use of supplemental imaging modalities has been shown to be advantageous in the assessment accuracy for dense breast tissue, and there are clear advantages and disadvantages to each type of diagnostic imaging test.

Awareness is increasing amongst the public and medical professionals regarding breast density as a risk factor for breast cancer, as well as the limitations of mammography in women with dense breasts. With this awareness comes legislation and notification laws in relation to breast density,^36^ particularly in countries with national screening protocols and programmes.

South Africa does not have a national screening programme for breast cancer, nor notification laws in connection to breast density. Across South Africa, in both the public and the private sectors of health care, there is a large discrepancy in breast imaging availability and quality. According to the South African National Health Policy document of 2017, screening mammography should not be introduced unless resources are available to ensure effective and reliable screening of at least 70% of the target group. Lack of resources and infrastructure in the South African public health care system renders a national screening programme untenable.^1^ Although neither clinical breast examination (CBE) nor breast self-examination (BSE) has yet to be established as screening tools, the utility of these interventions in limited resource areas is advocated by this policy document, as they promote breast health awareness. Breast self-examination as part of breast health awareness has been advocated for early detection in low-resource settings.^1^ Unfortunately, breast density cannot be determined by touch, by feel or by the appearance of a breast during the physical examination. It can only be determined by evaluating a mammogram.

Understanding breast cancer risk conferred by density in the setting of a patient’s history, as well as an appreciation of the imaging tools available, will help aid clinicians in developing the most appropriate screening plan for each of their patients. Mammography remains the most appropriate modality for population-based screening^37^ with the addition of one or more of the supplemental imaging modalities according to the patient’s individual breast cancer risk profile.^36^
